# Predictors of successful expectant and medical management of miscarriage: A systematic review

**DOI:** 10.1111/aogs.14934

**Published:** 2024-08-09

**Authors:** Sughashini Murugesu, Emily Braun, Srdjan Saso, Tom Bourne

**Affiliations:** ^1^ Queen Charlotte's and Chelsea Hospital Imperial College London UK; ^2^ Department of Metabolism, Digestion and Reproduction Imperial College London UK; ^3^ Department of Obstetrics and Gynaecology University Hospitals Leuven Leuven Belgium

**Keywords:** early pregnancy, expectant management, medical management, miscarriage, miscarriage management

## Abstract

**Introduction:**

15.3% of pregnancies result in miscarriage, management options include expectant, medical, or surgical. However, each patient has a range of variables, which makes navigating the available literature challenging when supporting individual patient decision‐making. This systematic review aims to investigate whether there are any specific predictors for miscarriage management outcome.

**Material and Methods:**

The following databases were searched, from the start of each database up to April 2023: PubMed, Medline, and Google Scholar. Inclusion criteria were studies interrogating defined predictors for expectant or medical management of miscarriage success. Exclusion criteria were poor quality, review articles, trial protocols, and congress abstracts. Data collection was carried as per PRISMA guidelines. Quality assessment for each study was assessed using the QUIPS proforma.

**Results:**

Relevant predictors include demographics, ultrasound features, presenting symptoms, and biochemical markers. Across the 24 studies there is heterogeneity in miscarriage definition, predictors reported, and management outcomes used. Associations with certain variables and miscarriage management outcomes are described. Ten studies assessed the impact of miscarriage type on expectant and/or medical management. The majority found that a diagnosis of incomplete miscarriage had a higher success rate following expectant or medical management compared to missed miscarriage or anembryonic pregnancy.

**Conclusions:**

We conclude that there is evidence supporting the possibility to offer personalized miscarriage management advice with case specific predictors. Further larger studies with consistent definitions of predictors, management, and outcomes are needed in order to better support women through the decision‐making of miscarriage management.

## Graphical abstract

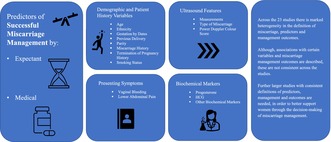

**Abbreviation**
HCG: human chorionic gonadotrophin

**Key message**

This is the first systematic review to address predictors of the success of expectant and medical management of miscarriage. There is evidence supporting the possibility to offer personalized miscarriage management advice with case specific predictors.

## 1 INTRODUCTION

There is a 15.3% overall risk of miscarriage in all recognized pregnancies.^1^ This translates to approximately 23 million miscarriages each year worldwide, or 44 each minute. Approximately half of women experiencing a miscarriage have been shown by ultrasonography at the time of diagnosis to have pregnancy tissue remaining within their uterus (missed or incomplete miscarriage). These patients then have to consider different options to manage their miscarriage: expectant, medical, or surgical.

Expectant management is advised as the first‐line recommendation for the majority of women in the National Institute for Care and Health Excellence guideline,^2^ leading to complete miscarriage in approximately 50% of women. This can continue safely for as long as necessary as per patient preference, provided that there is no evidence of infection.^3^ Expectant management is relatively inexpensive and safe, but its failure rate varies in the literature between 10% and 75%.^3^, ^4^, ^5^ Medical management is the alternative intervention to surgery. The success rate of medical management in studies varies between 69%^6^ and 80%.^7^ A challenge in the decision to opt for expectant or medical management is the lack of defined features that reliably predict the likelihood of complete resolution of the pregnancy.

The choice of miscarriage management is guided by patient preference, unless there is a clear indication for surgical intervention, such as hemorrhage or sepsis. The likely success rate has a significant influence on the approach chosen by individuals.^8^ A study by Graziosi et al.,^9^ found most women with a miscarriage would opt for medical treatment if the rate of complete resolution were greater than 65%. There is an emphasis in clinical practice on an informed decision‐making process, which for miscarriage management involves informing women of the chances of success in the context of all available information.^10^, ^11^

Established features that demonstrate an increased likelihood of success of expectant or medical management would be useful in the decision‐making process and may alleviate some of the established psychological burden of miscarriage.^12^ We present the results of a systematic review of studies investigating the clinical features that are predictive of successful expectant or medical management of missed or incomplete miscarriage.

## 2 MATERIAL AND METHODS

This study was conducted according to Preferred Reporting Items for Systematic Reviews and Meta‐Analyses (PRISMA) guidelines^13^ and registered with the international prospective register of systematic reviews PROSPERO under the registration number: CRD42022333397. No amendments were made to the protocol after the registration other than the use of an alternative bias evaluation tool (described below) and extension of the search date up to 2nd April 2023.

A search was performed for articles published up to 2nd April 2023 within several data bases (PubMed, Medline, and Google Scholar) to ensure all relevant comparative studies evaluating predictors of miscarriage expectant and medical management outcomes were identified.

A bibliographic search of English language publications in the computerized database PubMed was conducted. PubMed was our primary database where controlled vocabulary (Medical Subject Headings (MeSH) words) was used separately and in combination.^14^ Free text words were also used on PubMed and on the supplementary databases: Medline and Google Scholar.

**FIGURE 1:**
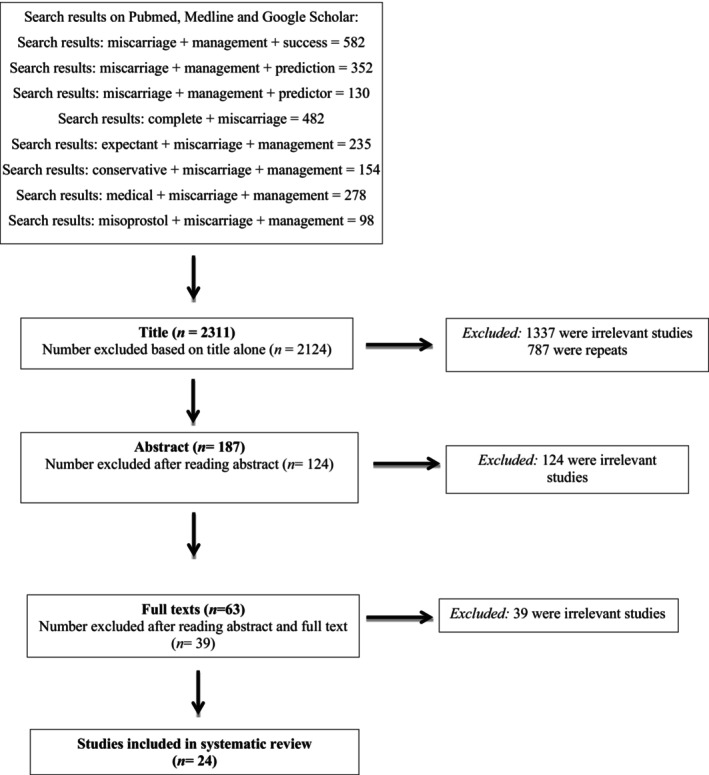
PRISMA flowchart.

The search terms, MeSH words, and combinations of searches used are outline in Figure 1. The search was augmented with the aid of a snowball strategy, which involved examining the references cited in primary sources and review manuscripts identified by the initial search. The screening and selection process of the relevant studies conducted is shown in Figure 1.

### 2.1 Study selection

Search results were screened in stages by title, abstract, and full text based on the following inclusion criteria: studies interrogating defined predictors for expectant or medical management of miscarriage success. All English language, peer‐reviewed human studies were included. Relevant studies published up to 2nd April 2023 were considered with no restrictions to the publication year. Exclusion criteria were: poor quality (eg inconsistent reporting of results), review articles, trial protocols, and congress abstracts.

### 2.2 Study screening

The initial search for the relevant studies was performed by the first author (SM) and was independently repeated by the second author (EB). After duplicate articles were removed, search results were screened consecutively by title, abstract, and full text as per the protocol. An overview of the search results and screening process is summarized in the study flow diagram (Figure 1). The screening process undertaken by the primary author and members of the project team was crosschecked by a senior author. Disagreement between the reviewers was resolved by discussion until consensus was reached.

### 2.3 Data extraction and analysis

A data extraction spreadsheet was developed and agreed between the authors. The selected studies were comprehensively examined. Relevant data were extracted for each paper and inputted into the spreadsheet by the first author (SM) and subsequently crosschecked by the second author (EB). Data were then analyzed qualitatively and summarized in the Section 3. Given the heterogeneity of the studies describing different predictors for successful outcomes following expectant or medical management of miscarriage, it was not possible to pool data together and perform a meta‐analysis. The authors of the selected studies were not contacted to provide any information other than what was presented in the studies. Quality assessment for each study was assessed using the QUIPS (Quality in Prognosis Studies) proforma. Disagreement regarding extracted data was resolved by discussion and deliberation by a more senior author (SS).

## 3 RESULTS

The results are presented in the following tables: Table 1 lists the duration of the study, number of patients, miscarriage type and management evaluated, aim, definition of successful management, results, and conclusions; Table 2 contains the predictive features for the outcome of each management option evaluated; Table 3 lists the inclusion and exclusion criteria for each study; Table 4 presents the QUIPS tool quality assessment of each included study.

**TABLE 1:** Miscarriage management prediction study characteristics and findings.

Author, year	Duration of study	Number of patients	Type of miscarriage	Miscarriage management	Study design	Aim	Outcome success definition	Results	Conclusion
Country									
Acharya, 2002 UK	Feb 2000–Jan 2001	86 patients	First Trimester Missed Miscarriage	Expectant management 4 weeks	Prospective Observational Cohort Study	To investigate whether gestational sac volume (GSV) can predict the outcome of missed miscarriages that are managed expectantly	Complete miscarriage defined as maximum anteroposterior endometrial diameter of less than 15 mm on transvaginal sonography and no persistent heavy vaginal bleeding	Logistic regression analysis showed no significant correlation between GSV and the outcome of missed miscarriages managed expectantly (*p* = 0.59)	GSV does not predict the outcome of expectant management of missed miscarriage within 4 weeks of the diagnosis
Banerjee, 2013 UK	2005–2007	52 patients	Missed Miscarriage <12 weeks	Medical management (Mifepristone and misoprostol regime), 3 days	Prospective Observational Cohort Study	To determine the relationship between serum progesterone level and the outcome of mifepristone misoprostol regimen for medical management of missed miscarriage up to 12 weeks	Success if retained tissues were expelled within 72 h	Serum progesterone between the successful and unsuccessful medical management were statistically significant (*p* = 0.001), by Mann–Whitney test	Mifepristone‐misoprostol regimen is less effective in missed miscarriage when serum progesterone is <10 nmol/L
Casikar, 2013 Australia	Nov 2006–July 2008	210 patients	First‐trimester miscarriage: incomplete, missed, and empty sac miscarriage	Expectant management 2 weeks	Prospective Observational Cohort Study	To evaluate whether symptomatology at presentation correlates with successful expectant management of first trimester miscarriage	Complete resolution of the miscarriage was defined as the resolution of symptoms, that is, cessation of vaginal bleeding and the absence of RPOC on follow‐up TVS	The overall rates of successful Expectant management was significantly different between the symptomatic (79.1%) and asymptomatic (42.9%) groups (*p*‐value = 0.0003). In the incomplete miscarriage group, higher rates of success were associated with vaginal bleeding than without (89.4 vs. 56.3%; *p*‐value = 0.0027). The presence or absence of pain was not found to be significant	Overall, vaginal bleeding at presentation was associated with an increase in success of expectant management; but individually, only significant in the incomplete miscarriage group. The presence or absence of pain at presentation was not a predictor for the various types of miscarriage
Casikar, 2013b Australia	Nov 2006–July 2009	186 patients training set 126 patients test set	First‐trimester miscarriage: incomplete, missed, and empty sac miscarriage	Expectant management 2 weeks	Prospective Observational Cohort Study	To generate and evaluate a new logistic regression model for the prediction of successful expectant management of first trimester miscarriage	Complete resolution of the miscarriage was defined as the resolution of symptoms, that is, cessation of vaginal bleeding and the absence of RPOC on follow‐up TVS	The most important Independent prognostic variables for the MLR model were as follows: type of miscarriage at primary scan, vaginal bleeding and maternal age. When developed retrospectively on a training data set, MLR model gave an area under the ROC curve (AUC) of 0.796. Prospective validation of MLR model on a new test data set resulted in an AUC of 0.803	Development and validation of mathematical model to predict successful management of first trimester miscarriage. Variables included: type of miscarriage at primary scan, vaginal bleeding and maternal age
Casikar, 2010 Australia	Nov 2006–July 2008	203 patients	First‐trimester miscarriage: incomplete, missed, and empty sac miscarriage	Expectant management 2 weeks	Prospective Observational Cohort Study	To assess uptake and success of expectant management of first‐trimester miscarriage for a finite 14‐day period, in order to evaluate the ‘2 week rule’ of management	Complete resolution of the miscarriage was defined as the resolution of symptoms, that is, cessation of vaginal bleeding and the absence of RPOC on follow‐up TVS	Overall spontaneous resolution of miscarriage at 2 weeks was observed in 61% (124/203) of women. Rates of spontaneous resolution at 2 weeks according to the type of miscarriage were 71% for incomplete miscarriage, 53% for empty sac and 35% for missed miscarriage	Expectant management based on the 2‐week rule is a viable and safe option for women with first‐trimester miscarriage. Women with an incomplete miscarriage are apparently the most suitable for expectant management
Casikar, 2012 Australia	Nov 2006–Feb 2009	158 patients	Incomplete miscarriage	Expectant management 2 weeks	Prospective Observational Cohort Study	To evaluate whether the use of subjective qualitative power doppler color scoring (PDCS) to confirm the presence or absence of blood flow within retained products of conception (RPC) in women with an incomplete miscarriage can predict subsequent successful expectant management.	Complete resolution of the miscarriage was defined as the resolution of symptoms, that is, cessation of vaginal bleeding and the absence of RPOC on follow‐up TVS	In the prediction of success, the absence of flow showed a sensitivity, specificity, positive predictive value, negative predictive value and positive likelihood ratio of 90.3, 37.5, 89, 40.9% and 1.445 (95% confidence interval: 1.055–1.979), respectively. There was no correlation between the volume of RPC and the PDCS; and there was no relationship between the volume of RPC and the success of expectant management	PDCS can predict the likelihood of successful expectant management of incomplete miscarriage. The absence of flow on Power Doppler is associated with a significant improvement in the rate of successful expectant management. This new approach may be helpful in quantifying the chances of successful expectant management in those women with an incomplete miscarriage at the primary scan
Creinin 2006 USA	Mar 2002–Mar 2004	485 patients	Missed Miscarriage, Incomplete/Inevitable Miscarriage	Medical (Misoprostol) Management, 30 days	Secondary Analysis of the medical management cohort in a RCT	To identify potential predictors for treatment success in medical management with misoprostol for early pregnancy failure	Misoprostol success was defined as complete miscarriage without the need for a vacuum aspiration within 30 days of treatment	Lower abdominal pain (*p* = 0.08) or vaginal bleeding within the last 24 h (*p* = 0.018), Rh‐negative blood type (*p* = 0.042), and nulliparity were predictive of overall success (parity *p* = 0.013). However, only vaginal bleeding within the last 24 h and parity of 0 or 1 were predictive of first‐dose success Multivariate analysis: Overall success exceeds 92% in women who have localized abdominal pain within the last 24 h, Rh‐negative blood type, or the combination of vaginal bleeding in the past 24 h and nulliparity	Misoprostol treatment for early pregnancy failure is highly successful in select women, primarily those with active bleeding and nulliparity. Clinicians and patients should be aware of these differences when considering misoprostol treatment
Elson, 2005 UK	Not specified	54 patients	Missed miscarriage and incomplete miscarriage	Expectant Management, follow up until resolution	Prospective Observational Cohort Study	To examine the value of various ultrasound and biochemical parameters for the prediction of successful expectant management of miscarriage	Expectant management was classified as successful if a complete miscarriage occurred without the need for surgical intervention	The size of retained products, serum HCG, progesterone, inhibin A and inhibin pro a‐C RI were all significantly different in those pregnancies that resolved spontaneously (*p* < 0.05) Serum inhibin A was the best predictor of a complete miscarriage	This study shows that novel Biochemical markers may be used to predict the likelihood of successful expectant management of miscarriage
Fernlund, 2020 Sweden	Sept 2008 – Dec 2015	83 patients expectant management 91 patients medical management misoprostol	Anembryonic or embryonic missed miscarriage	Expectant management. Two groups: ≤10 days ≤17 days Medical (Misoprostol) management. Two groups: ≤10 days ≤17 days	Prospective Observational Cohort Study	To identify predictors of complete miscarriage after expectant management or misoprostol treatment of non‐viable early pregnancy in women with vaginal bleeding	Complete evacuation on USS follow‐up	In multivariable logistic regression, the likelihood of success increased with increasing gestational age, increasing crown‐rump‐length and decreasing gestational sac diameter. Misoprostol treatment was successful in 80% (73/91). No variable predicted success of misoprostol treatment	Complete miscarriage after expectant management is significantly more likely in embryonic miscarriage than in anembryonic miscarriage. Gestational age, crown‐rump‐length, and gestational sac diameter are independent predictors of success of expectant management No variable predicted success of misoprostol treatment
Grewal, 2019 UK	June 2015 to June 2018	31 patients	First trimester fetal losses	Expectant Management, follow up until resolution	Prospective Observational Cohort Study	To report the incidence of enhanced myometrial vascularity (EMV) in consecutive women attending our early pregnancy assessment unit, following first‐trimester miscarriage. We aimed further to evaluate the clinical presentation and complications associated with expectant and surgical management of EMV in these women	Absence of RPOC	Expectant management was a safe option in patients with EMV with minimal bleeding, although it was associated with protracted time to resolution	This study suggests that EMV is an uncommon finding following miscarriage and is associated with the presence of RPOC. Expectant management was a safe option
Guedes‐Martins, 2015 Portugal	Jan 2011–March 2013	315 patients	Anembryonic or embryonic missed miscarriage	Medical management (Mifepristone and misoprostol regime), 8 weeks follow‐up	Prospective Observational Cohort Study	To determine the sensitivity and specificity of the uterine artery pulsatility (PI) and resistance (RI) indices to detect early pregnancy loss patients requiring dilation and curettage after unsuccessful medical management	Criteria for complete miscarriage included no evidence of retained products of conception in the uterine cavity and endometrial thickness (ET) <12 mm	The cutoff points for the uterine artery PI and RI, leading to the maximum values of sensitivity (69.5%, 95% CI: 61.5 to 76.5 and 75.0%, 95% CI: 57.9 to 86.8, respectively) and specificity (75.0%, 95% CI: 57.9 to 86.8 and 65.6%, 95% CI: 48.3 to 79.6, respectively), for the discrimination between the women who needed curettage from those who resolved spontaneously were 2.8 and 1, respectively	The potential usefulness of uterine artery Doppler evaluation to predict the need for uterine curettage in patients submitted to medical treatment for early pregnancy loss was demonstrated
Hamel 2022 Netherlands	Jun 2018‐ Jan 2020	344 patients	Women with a non‐viable pregnancy between 6 and 14 weeks of gestational age	Medical Management (Misoprostol +/− mifepristone), 6–8 week follow‐up	Secondary analysis of RCT	To determine the clinical predictors for successful medical management in cases of early pregnancy loss	The primary outcome was complete evacuation, defined as loss of the gestational sac and an endometrial thickness of <15 mm, at the latest 6–8 weeks after treatment start	Univariate analysis of possible predictors: Diagnosis *p* = 0.191, previous uterine aspiration *p* = 0.206. Multivariate model developed: The model includes the following variables: use of mifepristone, BMI, number of previous uterine aspirations, and the presence of minor clinical symptoms (slight vaginal bleeding or some abdominal cramps) at treatment start. The model shows a moderate capacity to discriminate between success and failure of treatment, with an AUC of 67.6% (95% CI 64.9 to 70.3)	Developed a prediction model aimed to improve and personalize counseling for medical treatment of EPL by providing a woman with her individual chance of complete evacuation with medical management
Lavecchia 2015 Canada	Jan 2011‐Dec 2013	227 patients	Early pregnancy loss presenting to ED	Medical Management (Misoprostol), follow‐up until resolution	Retrospective Cohort Study	To evaluate uterine content sonographic measurements for predicting medical management failure in early pregnancy loss	Outcomes of interest were defined as the need for dilation and curettage (D&C) and an unplanned return to the ED	Of all measurements evaluated, the cavity anteroposterior distance was found to be independently associated with D&C and an unplanned return to the ED. When a cavity anteroposterior distance cutoff of 15 mm was used, women were more likely to require D&C (adjusted odds ratio, 2.65; 95% confidence interval, 1.31–5.36; *p* < 0.01) and to have an unplanned return to the ED (adjusted odds ratio, 2.59; 95% confidence interval, 1.41–4.79; *p* < 0.01)	Patients identified as having a cavity anteroposterior distance of less than 15 mm should be considered good candidates for successful medical management
Luise, 2002 UK (BMJ)	Not specified	451 patients	Complete, incomplete, missed, or anembryonic	Expectant management, 4 weeks	Prospective Observational Cohort Study	To evaluate the uptake and outcome of expectant management of spontaneous first trimester miscarriage in an early pregnancy assessment unit	Complete miscarriage: the absence of transvaginal bleeding and an endometrial thickness <15 mm	A successful outcome without surgical intervention was seen in 81% of cases (367/451). The rate of spontaneous completion was 91% (201/221) for those cases classified as incomplete miscarriage, 76% (105/138) for missed miscarriage, and 66% (61/92) for anembryonic pregnancy. 70% of women completed their miscarriage within 14 days of classification (84% for incomplete miscarriage and 52% for missed miscarriage and anembryonic pregnancy)	Most women with retained products of conception chose expectant management Ultrasonography can be used to advise patients on the likelihood that their miscarriage will complete spontaneously within a given time
Luise, 2002 UK (UOG)	Not specified	221 patients	Incomplete miscarriage	Expectant management, 4 weeks	Prospective Observational Cohort Study	To assess whether the presence of a gestational Sac, or the width of the endometrium, can be used to predict the outcome of expectant management for an incomplete, first‐trimester miscarriage and to determine an appropriate schedule for follow‐up visits	Spontaneous completion of the miscarriage without surgical intervention: defined as an endometrial thickness <15 mm with no evidence of retained conception products and the absence of transvaginal bleeding and lower abdominal pain	201 (91%) completed their miscarriage without intervention; the mean time from diagnosis to completion was 9 (range, 1–32) days. By the end of week 2, 184 women (83%) had miscarried. There was no statistically significant relationship between the initial presence of a gestational sac or endometrial thickness and the success rate of expectant management	Most women with an incomplete, spontaneous Miscarriage chose expectant management and had a successful outcome. Neither the presence of a gestational sac nor the endometrial thickness at diagnosis can be used to predict the likelihood of management failure
Lusink, 2018 Australia	3 year period	296 patients	Missed or Incomplete Miscarriage	Medical Management (misoprostol), 2 weeks	Retrospective cohort study	To determine factors predictive of successful medical management, utilizing a single dose protocol of 800 μg vaginal misoprostol	Success was defined as an empty uterus with an endometrial thickness of <15 mm at transvaginal ultrasound scan	In this cohort, the success rate was 67% (199/296), and smaller mean gestational sac diameter (MGSD) independent of gestational age predicted success (*p* = 0.046). Success is not significantly related to parity, miscarriage type, pelvic pain, or vaginal bleeding at the outset of treatment	This study suggests that larger MGSD has lower odds of success when controlled for gestational age. Predictive modeling may be possible in larger studies so that women can be given more information about their individual likelihood of success with medical management of miscarriage
Sairam, 2001 UK	12‐month period	305 patients	Missed or Incomplete Miscarriage	Expectant management, 3 weeks	Prospective Observational Cohort Study	To define the sonographic criteria that best determine the likelihood of successful expectant management of early pregnancy failure (EPF)	Expectant management of early pregnancy loss was deemed to have failed if surgical evacuation was performed because of prolonged or heavy bleeding, excessive abdominal pain, suspected endometritis, ultrasound evidence of retained products (day 21), positive ßhCG (day 21), or if the woman chose to have surgery	The success rate for incomplete miscarriage (96%) was significantly better than that for missed miscarriage (62%)	Ultrasound has an invaluable role in predicting the likelihood of successful expectant management enabling patients to make an informed choice about their medical care
Sajan, 2020 India	3 years	100 patients	Three groups of early pregnancy miscarriages: anembryonic pregnancy, early fetal demise, and incomplete miscarriage	Expectant management, 2 weeks	Prospective Observational Cohort Study	To compare success rate and complications in expectant management in three groups of early pregnancy miscarriages‐ incomplete miscarriage, anembryonic pregnancy, and early fetal demise (EFD)	Criteria for complete miscarriage: USG endometrial thickness of less than 15 mm measured in the anteroposterior plane associated with the cessation of heavy bleeding and pain	Incomplete miscarriage group had highest success rate of 88.46%. followed by anembryonic pregnancy (72.5%) and EFD (47.83%) *p* value = 0.007. Complication rate was highest in EFD, followed by anembryonic, and the least in incomplete miscarriage all of which was statistically significant except vaginal bleeding	Expectant management should be offered as first line choice for all types of early pregnancy miscarriages. Proper selection of cases as to type of miscarriage, especially incomplete miscarriage, and selected cases of anembryonic pregnancy and EFD ensures higher success rate with lesser complications. Reserving medical and surgical management for unsuitable/failed cases
Schreiber, 2015 USA	2002–2004	95 patients	First trimester pregnancy failure: anembryonic pregnancy, embryonic demise, incomplete miscarriage, and inevitable miscarriage	Medical Management (Misoprostol +/− mifepristone), 2 weeks follow‐up	Sub‐analysis of data collected as part of a randomized controlled, multicenter trial	To elucidate predictors of successful medical management of miscarriage with a single dose of misoprostol	If the sonographic and clinical evaluation were consistent with complete expulsion of the products of conception	A multivariable logistic model for success included non‐Hispanic ethnicity and parity <2 in addition to hCG ≥4000 mIU/mL and ADAM‐12 ≥2500 pg/mL and had an area under the receiver operating characteristic (ROC) of 0.81 (95% CI: 72 to 90). Categorizing women with a predicted probability of ≥0.65 resulted in a sensitivity of 75.0%, specificity 77.1%, and positive predictive value of 81.8%	While preliminary, the data suggest that serum biomarkers, especially when combined with demographic characteristics, may be helpful in guiding patient decision‐making regarding the management of early pregnancy failure. Further study is warranted
Schwarzler, 1999 UK	Not specified	85 patients	Missed miscarriage and anembryonic pregnancy	Expectant Management, 4 weeks	Prospective Observational Cohort Study	Study of patients with first trimester miscarriage evaluates whether conservative management is a feasible strategy and assesses the value of color Doppler ultrasonography for patient selection	Complete miscarriage was defined as having an endometrial cavity thickness of <10 mm and a negative urinary pregnancy test (Clearview, 50 IU/L; Unipath Ltd. Bedford, UK) or serum HCG value of <50 IU/L	Of patients in the conservative management group, 71 out of 85 (84%) had a spontaneous, complete miscarriage, while 37 out of 46 cases (80%) with detectable presumed intervillous pulsatile blood flow had a complete, spontaneous miscarriage within 1 week; this occurred in 23% of cases with no detectable flow	This suggests that conservative management is a successful approach for many patients with first trimester miscarriage; color Doppler ultrasonography can be used to select the most suitable patients for this strategy and thus reduce the need for hospital admission and surgery
Sonalkar, 2021 USA	Not specified	297 patients	Anembryonic gestation or embryonic/fetal demise	Medical Management (Misoprostol +/− mifepristone), 30 days follow‐up	Secondary analysis of data collected as part of a randomized trial	To evaluate characteristics associated with treatment success in women receiving medical management with mifepristone misoprostol or misoprostol alone for early pregnancy loss (EPL)	The primary outcome was complete expulsion of the gestational sac by the first follow‐up visit (24 h after misoprostol use, range days 2–5) without further intervention over the 30‐day study period	In the full cohort, the only significant predictors of treatment success were mifepristone pretreatment (aOR 2.51, 95% CI 1.43 to 4.43) and smoking (aOR 2.15, 95% CI 1.03 to 4.49)	No baseline clinical factors predict success in women undergoing medical management of EPL with misoprostol. Adding mifepristone to the EPL medical management regimen improves treatment success and should be used regardless of baseline clinical characteristics
Trinder, 2006 UK	May 1997–Dec 2001	1200 patients	Early fetal demise or incomplete miscarraige	Expectant Management and Medical management (Mifepristone and misoprostol regime) To resolution, up to 8 weeks	Randomized Controlled Trial	Randomized controlled trial comparing medical and expectant management with surgical management of first trimester miscarriage	Failure defined as need for unplanned admission or surgical intervention	Expectant Management: Early fetal demise 50% required surgery, incomplete miscarriage 25% required surgery later Medical Management: Early fetal demise 38% required surgery, incomplete miscarriage 29% required surgery later	Outcomes of both expectant and medical management differed depending on miscarriage type
Vejborg, 2007 Denmark	Dec 2001–Aug 2003	355 patients	Missed miscarriage or anembryonic pregnancy	Medical Management (Misoprostol), 3 days	Retrospective cohort study	To assess if a single dose of misoprostol could reduce the number of surgical interventions in early pregnancy failure, and to compare efficacy in different ultrasonographically‐defined subgroups	A complete miscarriage was defined as no gestational sac, largest anteroposterior endometrial diameter </=15 mm, and if the doctor had found the vaginal bleeding to be acceptable	The regimen was more efficacious in missed miscarriage CRL <6 mm group (50%) and less efficacious in anembryonic pregnancy with gestational sac >/=18 mm (26.6%) than in the other groups. The success rates were lower 1 day after treatment (30.2%) compared to days 2 and 3 (43.6%; pB0.05), and the difference was largest in Group B1 (12.9 vs. 35.4%; pB0.05)	The success rates of medically treated first trimester miscarriages varied according to the ultrasonographic definitions of pregnancy failure, time of assessment, and the criteria for success
Wada, 2021 Japan	Jan 2011–Aug 2019	44 patients	Retained products of conception following miscarriage	Expectant Management, 2 weeks	Retrospective cohort study	To clarify the natural history of retained products of conception (RPOC) following miscarriage at less than 22 weeks of gestation and those who show major bleeding during course observation	Checked intrauterine findings including the presence or absence of RPOC at 0–1 and 7–14 days following miscarriage	Of the 44, 34 (77%) patients were observed without intervention (recovery group); the other 10 (23%) patients required additional interventions associated with subsequent bleeding (intervention group). Compared with the recovery group, heavy bleeding (>500 mL) at miscarriage (6/10: 60%) and RPOC hypervascularity (8/10: 80%) were more frequently observed in the intervention group	Expectant management was successful in almost 80% of patients with RPOC following miscarriage. The additional interventions were required in patients with heavy bleeding at miscarriage and RPOC hypervascularity

Abbreviations: aOR, adjusted odds ratio; AUC, area under the ROC curve; BMI, body mass index; CI, confidence interval; CRL, crown rump length; D&C, dilatation and curretage; ED, emergency department; EFD, early fetal demise; EMV, enhanced myometrial vascularity; EPF, early pregnancy failure; EPL, early pregnancy loss; GSV, gestational sac volume; MGSD, mean gestational sac diameter; MLR, multiple linear regression; PDCS, power Doppler colour scoring; PI, pulsatility index; RCT, randomised controlled trial; RI, resistance index; ROC, receiver operator characteristic curve; RPC, retained products of conception; RPOC, retained products of conception.

**TABLE 2:** Miscarriage features analyzed.

*Demographic and patient history variables*		
Casikar, 2013b Australia	Student *t*‐tests to compare features between patients with success and failure of expectant management	
	Training Set *p*‐values: *Maternal Age (year)* 0.0250 Gestation (day) 0.0035 NVD History 0.0869 LSCS History 0.8072 *Miscarriage History* 0.0349 TOP History 0.6614 Smoker 0.1058	Test Set *p*‐values: *Maternal Age (year)* 0.0110 Gestation (day) 0.7720 NVD History 0.2847 LSCS History 1 Miscarriage History 0.1946 TOP History 1 Smoker 0.3093
	Maternal age included in multinominal logistic regression (MLR) model	
Creinin, 2006 USA	Successful *n* = 410, Failure *n* = 75. *Nulliparity* was predictive of overall success (parity *p* = 0.013). *Rh‐negative blood type* predictive of success (*p* = 0.042)	
Elson, 2005 UK	*p*‐Values comparing successful (*n* = 37) and unsuccessful (*n* = 17) expectant management groups Maternal age: >0.05 Gestational age: >0.05	
Fernlund, 2020 Sweden	*p*‐Values comparing successful (*n* = 39) and unsuccessful (*n* = 46) expectant management ≤10 days groups Gestational age by LMP: 0.053 Vaginal del: 0.374 Parity: 0.887 *p*‐Values comparing successful (*n* = 44) and unsuccessful (*n* = 39) expectant management ≤17 days groups Gestational age by LMP: 0.04 Vaginal del: 0.843 Parity: 0.679 No variable predicted success of misoprostol treatment either in embryonic or anembryonic miscarriages.	
Hamel, 2022 Netherlands	Success *n* = 237, Unsuccessful *n* = 107 Univariate analysis Ethnicity *p* = 0.667 Previous miscarriage *p* = 0.992 Previous medical management of miscarriage *p* = 0.545 Previous uterine aspiration *p* = 0.206 Prediction model included: *BMI* regression coefficient 0.077 *Number of previous uterine aspirations* regression coefficient − 0.501	
Lusink, 2018 Australia	Successful medical management of miscarriage: *p*‐value Parity: 0.63 Time delay to misoprostol: 0.65	
Schreiber, 2015 USA	Successful medical management of miscarriage: *p*‐value Maternal Age: 0.902 Race: 0.505 *Hispanic*: 0.009 *BMI*: 0.009 Number of prior miscarriages: 0.692 *Parity*: 0.010 Type of pregnancy: 0.545	
Schwarzler, 1999 UK	The chances of the miscarriage resolving were independent of gestational age	
Sonalkar, 2021 USA	We found no clinical or medical history predictors of medical management success, except for *non‐smoking status* (*p* = *0.01*) Successful medical management of miscarriage: *p*‐value Parity 0.27 Median maternal age 0.5 Mean BMI 0.64 Race 0.60 Ethnicity Hispanic 0.51 Prior early pregnancy loss 0.87 Prior induced miscarriage 0.40 Prior medical miscarriage 0.23 Prior surgical miscarriage 0.21 Gestational age 0.75 Method of conception 0.13 Rh status 0.94	
*Ultrasound features*		
Acharya, 2002 UK	GSV (Gestational Sac Volume) – No correlation to predict outcome of expectant management within 4 weeks of miscarriage diagnosis	
Casikar, 2013b Australia	Student *t*‐tests to compare features between patients with success and failure of expectant management	
	Training Set *p*‐values: ET (mm) 0.9749 Log RPOC vol. 0.7178 Missed miscarriage 1.92 E‐05 Empty sac miscarriage 0.2471 Incomplete miscarriage 4.12 E‐06	Test Set *p*‐values: ET (mm) 0.7607 Log RPOC vol. 0.0643 *Missed miscarriage 0.0036* *Empty sac miscarriage 0.0003* *Incomplete miscarriage 6.47E‐07*
	Type of miscarriage included in MLR model	
Casikar, 2010 Australia	Rates of spontaneous resolution at 2 weeks according to the type of miscarriage were 71% for incomplete miscarriage, 53% for empty sac, and 35% for missed miscarriage.	
Casikar, 2012 Australia	*Qualitative power doppler color scoring (PDCS)*: In the prediction of successful expectant management, the absence of vascularity within the RPOC demonstrated a sensitivity of 90.3, specificity of 37.5%, positive predictive value of 89%, negative predictive value of 40.9%, and positive likelihood ratio of 1.445 (95% confidence interval: 1.055–1.979)	
Elson, 2005 UK	P‐values comparing successful (*n* = 37) and unsuccessful (*n* = 17) expectant management groups *Diameter of products of conception*: <*0.05*	
Fernlund, 2020 Sweden	P‐values comparing successful (*n* = 39) and unsuccessful (*n* = 46) expectant management ≤10 days groups Gestational sac: 0.624 Gestational sac diameter: 0.448 *CRL*: 0.008 Miscarriage type on US: 0.063 CRL if embryonic: 0.06 Blood flow in presrumed intervillous space by Gray‐scale US: 0.288 Color Doppler US: 0.109 Spectral Doppler US: 0.619 *p*‐Values comparing successful (*n* = 44) and unsuccessful (*n* = 39) expectant management ≤17 days groups Gestational sac: 0.925 Gestational sac diameter: 0.967 *CRL*: 0.004 *Miscarriage type on US*: 0.024 CRL if embryonic: 0.069 Blood flow in presrumed intervillous space by Gray‐scale US: 0.105 Color Doppler US: 0.008 Spectral Doppler US: 0.125 No variable predicted success of misoprostol treatment either in embryonic or anembryonic miscarriages.	
Grewal, 2019 UK	In all cases in this study cohort, the initial presence of *enhanced myometrial vascularity (EMV)* following first‐trimester miscarriage was associated with the presence of RPOC, and this was confirmed histologically in all surgically managed cases.	
Guedes‐Martins, Portugal 2015	The cut‐off points for *the uterine artery pulsatility (PI) and resistance (RI) indices*, leading to the maximum values of sensitivity (69.5%, 95% CI: 61.5 to 76.5 and 75.0%, 95% CI: 57.9 to 86.8, respectively) and specificity (75.0%, 95% CI: 57.9 to 86.8 and 65.6%, 95% CI: 48.3 to 79.6, respectively), for the discrimination between the women who needed curettage from those who resolved spontaneously were 2.8 and 1, respectively	
Lavecchia, 2015 Canada	P‐values comparing successful (*n* = 182) and unsuccessful (*n* = 45) (D&C needed) medical management groups Cavity transverse diameter: NS Cavity longitudinal distance: NS *Cavity anteroposterior distance*: <0.05 Volume: NS NS=Not significant	
Luise, 2002 UK (BMJ)	The overall rate of spontaneous completion for cases classified as incomplete miscarriage was 201/221 (91%); the value for missed miscarriage was 105/138 (76%) and for anembryonic pregnancies 61/92 (66%). Overall, 52% of incomplete miscarriages had resolved spontaneously by day 7 of management and 84% by day 14. The corresponding values for missed miscarriages and anembryonic pregnancies were 28% by day 7 and 56% by day 14.	
Luise, 2002 UK (UOG	A gestational sac was visible in 32% of cases and an endometrial thickness equal to or less than 15 mm in a further 38% of cases. There was no significant correlation between the presence or absence of a gestational sac and the proportion of completed miscarriages (Yates corrected chi‐square test, *p* > 1.0). Similarly, the trend towards a decrease in the proportion of completed miscarriages with the increase in endometrial thickness was not statistically significant (chi‐square test, *p* > 0.61)	
Lusink, 2018 Australia	Successful medical management of miscarriage: *p*‐Value *MGSD (mean gestation sac diameter)*: 0.046 Miscarriage type: 0.40	
Sairam, 2001 UK	The success rate in the group with *incomplete miscarriage* (96%) was significantly higher than that in the group with missed miscarriage (62%; OR, 1.5; 95% CI, 1.04 to 2.29)	
Sajan, 2020 India	*Incomplete miscarriage* group had highest success rate of 88.46%. followed by anembryonic pregnancy (72.5%) and EFD (47.83%) *p* value = 0.007	
Schreiber, 2015 USA	Successful medical management of miscarriage: *p*‐value Baseline gestational sac diameter: 0.613	
Schwarzler, 1999 UK	In the conservatively managed group, the presence of blood flow in the presumed intervillous space was associated with the likelihood of complete spontaneous miscarriage within 7 days. Of pregnancies with positive presumed intervillous flow, 80% underwent spontaneous resolution, whereas this only occurred in 23% of those where presumed intervillous flow was absent	
Sonalkar, 2021 USA	Successful medical management of miscarriage: *p*‐Value Diagnosis embryonic/fetal demise or anembryonic gestation 0.52	
Trinder 2006, UK	Expectant Management: Early fetal demise 50% required surgery, incomplete miscarriage 25% required surgery later Medical Management: Early fetal demise 38% required surgery, incomplete miscarriage 29% required surgery later	
Vejborg, 2007 Denmark	Highest success rate in Group: fetus <6 mm and no growth or development of cardiac activity over 1 week (50 vs. 36.2%; *p* < 0.05), and the lowest success rate in Group: anembryonic pregnancy with an empty gestational sac </=18 mm (26.6 vs. 42.8%; *p* < 0.01) compared to the other 3 groups.	
Wada, 2021 Japan	Compared with the recovery group, RPOC hypervascularity (8/10: 80%) was more frequently observed in the intervention group, respectively.	
*Presenting symptoms*		
Casikar, 2013 Australia	*Vaginal bleeding*: overall rates of successful expectant management were significantly higher in women with vaginal bleeding compared with those without any vaginal bleeding (*p* = 4.8 × 10^6^) Lower abdominal pain: Overall rates of successful expectant management were not significantly higher in women with absent lower abdominal pain compared with those with lower abdominal pain present (68.9% vs. 78.9%, *p*‐value = 0.1722).	
Casikar, 2013b Australia	Student *t*‐tests to compare features between patients with success and failure of expectant management	
	Training Set *p*‐values: Nil Vaginal Bleeding 4.81E‐06 Vaginal Bleeding without clots 0.4875 *Vaginal Bleeding with clots* 0.0014 Abdominal pain 0.0954 *Scan asymptomatic* 0.0003 VAS (0–100) 0.4107	Test Set *p*‐values: *Nil Vaginal Bleeding* 0.0002 Vaginal Bleeding without clots 0.6746 *Vaginal Bleeding with clots* 0.0129 Abdominal pain 0.1619 *Scan asymptomatic* 0.0002 VAS (0–100) 0.1986
	Vaginal Bleeding included in MLR Model	
Creinin, 2006 USA	Successful *n* = 410, failure *n* = 75. Lower abdominal pain (*p* = 0.08) or vaginal bleeding within the last 24 h (*p* = 0.018) were predictive of overall success	
Elson, 2005 UK	*p*‐values comparing successful (*n* = 37) and unsuccessful (*n* = 17) expectant management groups Vaginal bleeding: >0.05	
Fernlund, 2020 Sweden	*p*‐values comparing successful (*n* = 39) and unsuccessful (*n* = 46) expectant management ≤10 days groups Bleeding at inclusion: 0.218, Pain at inclusion: 0.633 *p*‐values comparing successful (*n* = 44) and unsuccessful (*n* = 39) expectant management ≤17 days groups Bleeding at inclusion: 0.053, Pain at inclusion: 0.893 No variable predicted success of misoprostol treatment either in embryonic or anembryonic miscarriages	
Hamel, 2022 Netherland	Success *n* = 237, Unsuccessful *n* = 107 Prediction model included: Minor clinical symptoms present at treatment start: regression coefficient 0.853	
Lusink, 2018 Australia	Successful medical management of miscarriage: *p*‐value Pain: 0.27 Bleeding: 0.90	
Schreiber, 2015 USA	Successful medical management of miscarriage: *p*‐value Duration of bleeding: 0.626 Lower abdo pain: 0.972 Vaginal bleeding: 0.131	
Sonalkar, 2021 USA	Successful medical management of miscarriage: *p*‐value Pain during periods 0.19 Active bleeding 0.74 Uterine tenderness 0.80	
Wada, 2021 Japan	Compared with the recovery group, *heavy bleeding* at miscarriage (6/10: 60%) were more frequently observed in the intervention group, respectively	
*Biochemical markers*		
Banerjee, 2013 UK	*Serum Progesterone* at the time of mifepristone administration. Serum progesterone level < 10 nmol/L (3 ng/L) is associated with failed mifepristone‐misoprostol regimen (*p* < 0 0.001)	
Elson, 2005 UK	*p*‐values comparing successful (*n* = 37) and unsuccessful (*n* = 17) expectant management groups *bHCG*: <*0.001* *progesterone*: <*0.05* 17‐aOH progesterone: >0.05 IGFBP‐1: >0.05 *Inhibin A*: <*0.001* *Inhibin pro aC‐RI*: <*0.05*	
Fernlund, 2020 Sweden	*p*‐values comparing successful (*n* = 39) and unsuccessful (*n* = 46) expectant management ≤10 days groups *bHCG*: 0.01 *Progesterone*: 0.011 *p*‐values comparing successful (*n* = 44) and unsuccessful (*n* = 39) expectant management ≤17 days groups *bHCG*: 0.01 *Progesterone*: 0.003 No variable predicted success of misoprostol treatment either in embryonic or anembryonic miscarriages.	
Schreiber, 2015 USA	Successful medical management of miscarriage: *p*‐value Activin A < 240: 0.351 Glycodelin < 3: 0.336 *ADAM‐12* < *2500*: 0.032 HPL <5: 0.059 Estradiol <500: 0.294 Progesterone <10: 0.424 hCG <4000: 0.061 Logistic predictive model *HCG* < *4000*: 0.029	
Schwarzler, 1999 UK	Logistic regression analysis to predict occurrence of spontaneous miscarriage: *p*‐value *Progesterone*: <0.001 *HCG*: 0.029	

Abbreviations: BMI, body mass index; CI, confidence interval; CRL, crown rump length; D&C, dilatation and curettage; ED, emergency department; EMV, enhanced myometrial vascularity; ET, endometrial thickness; GSV, gestational sac volume; LMP, last menstrual period; LSCS, lower segment cesarean section; MGSD, mean gestational sac diameter; MLR, multiple linear regression; NVD, normal vaginal delivery; PDCS, power Doppler color scoring; PI, pulsatility index; RI, resistance index; RPOC, retained products of conception; TOP, termination of pregnancy; TVS, transvaginal ultrasound scan; US, ultrasound.

Italics indicate predictors with a *p*‐value <0.5.

**TABLE 3:** Inclusion and exclusion criteria.

Author, year	Inclusion	Exclusion
Country		
Acharya, 2002 UK	All patients with a confirmed first‐trimester missed miscarriage who chose to undergo expectant management	
Banerjee, 2013 UK	Ultrasound confirmed missed miscarriage (crown‐rump length <6 mm with no fetal heart beat) or early fetal demise (gestational sac diameter >20 mm without fetal pole or yolk sac) Upper limit for gestational age was 12 weeks by menstrual date or by ultrasound scan	If active vaginal bleeding Previously received medical management for miscarriage in the current pregnancy
Casikar, 2013 Australia	Women diagnosed with incomplete, missed, and empty sac miscarriage were included An incomplete miscarriage was defined as the presence of heterogeneous tissue seen on ultrasound within the uterine cavity and distorting the endometrial midline echo Missed miscarriage was defined as the presence of a crown‐rump length (CRL) </=6 mm with absent fetal heart activity seen on ultrasound An empty sac was defined as a gestational sac diameter >20 mm with no visible CRL seen on ultrasound	Severe vaginal hemorrhage with hemodynamic instability, Infection Complete miscarriage diagnosed at the first scan (in the context of a previously documented intrauterine pregnancy) Molar pregnancy Missed miscarriage diagnosed at the nuchal translucency (NT) scan Missed miscarriage when the crown‐rump length (CRL) was 3 weeks smaller than the gestational age based on the last menstrual period (LMP)
Casikar, 2013 Australia	Women diagnosed with incomplete, missed, and empty sac miscarriage were included An incomplete miscarriage was defined as the presence of heterogeneous tissue seen on ultrasound within the uterine cavity and distorting the endometrial midline echo Missed miscarriage was defined as the presence of a crown‐rump length (CRL) </=6 mm with absent fetal heart activity seen on ultrasound An empty sac was defined as a gestational sac diameter >20 mm with no visible CRL seen on ultrasound	Severe vaginal hemorrhage with hemodynamic instability Infection Complete miscarriage diagnosed at the first scan (in the context of a previously documented intrauterine pregnancy) Molar pregnancy Missed miscarriage diagnosed at the nuchal translucency (NT) scan Missed miscarriage when the crown‐rump length (CRL) was 3 weeks smaller than the gestational age based on the last menstrual period (LMP)
Casikar, 2010 Australia	Women diagnosed with incomplete, missed, and empty sac miscarriage were included An incomplete miscarriage was defined as the presence of heterogeneous tissue seen on ultrasound within the uterine cavity and distorting the endometrial midline echo Missed miscarriage was defined as the presence of a crown‐rump length (CRL) </=6 mm with absent fetal heart activity seen on ultrasound An empty sac was defined as a gestational sac diameter >20 mm with no visible CRL seen on ultrasound	Severe vaginal hemorrhage with hemodynamic instability, Infection Complete miscarriage diagnosed at the first scan (in the context of a previously documented intrauterine pregnancy) Molar pregnancy Missed miscarriage diagnosed at the nuchal translucency (NT) scan Missed miscarriage when the crown‐rump length (CRL) was 3 weeks smaller than the gestational age based on the last menstrual period (LMP)
Casikar, 2012 Australia	Diagnosis of incomplete miscarriage: defined by the presence of a measurable focus of hyperechoic material within the endometrial cavity using two‐dimensional (2D) greyscale TVS Hemodynamic stability Absence of infection	Severe vaginal hemorrhage or hemodynamic instability Signs of infection: temperature, tachycardia, offensive vaginal loss and lower abdominal tenderness
Creinin, 2006 USA	Women who had an anembryonic gestation or embryonic/fetal demise were eligible for inclusion if they had an ultrasound examination demonstrating an embryonic pole or crown‐rump length between 5 and 40 mm without cardiac activity, an anembryonic gestational sac with a mean diameter between 16 and 45 mm, growth of gestational sac less than 2 mm over 5 days or less than 3 mm over 7 days, or an increase in human chorionic gonadotropin by less than 15% over 2 days with a yolk sac visualized by ultrasound examination Women who had incomplete or inevitable miscarriages were also included Incomplete spontaneous miscarriage was defined as passage of some products of conception with the residual anterior–posterior endometrial lining greater than 30 mm by transvaginal ultrasonography and uterine size less than 13 weeks. This cut‐off was based on evidence from prior studies of women treated with misoprostol for medical miscarriage or early pregnancy failure Inevitable miscarriage was defined as an intrauterine gestational sac less than 45 mm or embryonic pole less than 40 mm and an open internal cervical os to digital examination with active vaginal bleeding	Women were excluded from the primary study if they were: anemic (hemoglobin level below 9.5 mg/dL) hemodynamically unstable history of a clotting disorder using anticoagulants allergic to prostaglandins or nonsteroidal anti‐inflammatory drugs previously undergone surgical or medical miscarriage, either self‐induced or by other physicians during the current pregnancy
Elson, 2005 UK	A diagnosis of missed miscarriage was made if the size of gestational sac was >20 mm in diameter with no visible embryo; if the fetal crown–rump length was >5 mm with no detectable fetal heart rate; or if the gestational sac had failed to develop on a follow‐up scan >6 days after the initial examination An incomplete miscarriage was diagnosed in women with a history of bleeding, who had no visible gestational sac on ultrasound scan, but there was clear evidence of retained trophoblast tissue within the uterine cavity All clinically stable women with history of mild lower abdominal pain and bleeding and a conclusive ultrasound diagnosis of miscarriage were offered expectant management	
Fernlund, 2020 Sweden	≥18 years old understanding written and spoken Swedish hemoglobin concentration >80 g/L no contraindications to misoprostol treatment fulfilling ultrasound criteria of anembryonic or embryonic miscarriage fetal crown‐rump‐length ≤33 mm	Women with heavy bleeding needing urgent surgical evacuation of the uterine cavity, as judged clinically, were not eligible
Grewal, 2019 UK	Women presented after miscarriage with the following clinical scenarios: incomplete miscarriage (*n* = 23), failed medical management of miscarriage with RPOC (*n* = 8) and failed surgical management of miscarriage (*n* = 9) EMV was defined as a hypervascular area within the myometrium, detected on transvaginal color Doppler ultrasonography with a PSV on pulsed Doppler of ≥20 cm/s, within the collection of vessels	
Guedes‐Martins, Portugal 2015	Cases of early pregnancy loss, defined as intrauterine pregnancy with reproducible evidence of lost fetal heart activity and/or the lack of increased crown–rump length over one week or the persisting presence of an empty sac at less than 12 weeks of gestation Clinically stable women	Ectopic pregnancy Complete or incomplete pregnancy Known allergy to prostaglandins or NSAIDs Multiple gestation Heavy vaginal bleeding Hemodynamic instability Blood clotting problems or current treatment with anticoagulants Hb <10 Body temperature >38 degrees CRL >12 weeks gestation Prostaglandin contraindications, including uncontrolled blood pressure, mitral stenosis, severe asthma, or glaucoma
Hamel, 2022 Netherlands	Women aged 16 years or older Diagnosed with a non‐viable intrauterine pregnancy between 6 and 14 weeks of gestational age	Women who were clinically unfit for medical management Women with a miscarriage in progress (defined as increasing or heavy vaginal bleeding and/or abdominal cramping) or with an incomplete miscarriage
Lavecchia, 2015 Canada	ED discharge prescription for medical management	Women whose medical charts lacked their sonograms
Luise, 2002 UK (BMJ)	Women with retained products of conception opting for expectant management	Molar pregnancy
Luise, 2002 UK (UOG)	All women with a spontaneous, incomplete miscarriage before the 13th week of gestation	Molar pregnancy
Lusink, 2018 Australia	The crown rump length should be ≤25 mm and the MGSD ≤50 mm The woman should have no medical contraindications to misoprostol and have adequate social support at home	Received mifepristone Protocol violations Incomplete records
Sairam, 2001 UK	Incomplete miscarriage was diagnosed when the endometrial thickness was more than 5 mm with loss of midline echo suggestive of retained products. Missed miscarriage was diagnosed when either the mean diameter of the empty gestation sac was ≥20 mm (anembryonic gestation) or when the crown rump length was ≥7 mm without a detectable heart beat (missed miscarriage)	Heavy vaginal bleeding Severe abdominal pain Clinical evidence of intrauterine infection Suspected molar/ectopic pregnancy Other medical complications Gestation age greater than 14 weeks
Sajan, 2020 India	Anembryonic pregnancy: Gestational sac mean diameter of at least 25 mm with no embryonic/extra embryonic structures present Early fetal demise (EFD): CRL of at least 7 mm with no cardiac activity, or no change in size on weekly serial scanning Incomplete miscarriage: Disrupted endometrial echo measuring more than 15 mm measured in the anteroposterior plane, with the presence of heterogeneous and irregular tissues	Gestational age more than13 weeks Severe vaginal bleeding Severe abdominal pain Hemodynamic instability at presentation Fever Chronic illness
Schreiber, 2015 USA	Women presenting with first trimester pregnancy failure (anembryonic pregnancy, embryonic demise, incomplete miscarriage, and inevitable miscarriage)	
Schwarzler, 1999 UK	Pregnancies with an estimated gestation age of <13 weeks from the last menstrual period (LMP) were included. All women included in the study were symptomatic and had reported vaginal bleeding and/or abdominal pain. Those shown by ultrasonography to have either a missed miscarriage or an anembryonic pregnancy	Severe hemorrhage or pain Pyrexia above 37.5°C Severe asthma Hemolytic disease or blood dyscrasias Current anticoagulation or systemic corticosteroid treatment Twin or higher order pregnancy Smoker aged over 35 Inability to understand written English
Trinder, 2006 UK	Women with a pregnancy of less than 13 weeks' gestation Diagnosed as having either an incomplete miscarriage (defined an incomplete miscarriage as areas of mixed echogenicity within the uterine cavity with or without a disordered gestation sac) or early fetal/embryonic demise (defined early embryonic demise as an intact gestation sac of greater than 20 mm mean diameter with no other internal structures and early fetal demise as a fetus of over 6 mm crown rump length with no heart activity on transvaginal ultrasound scan)	
Sonalkar, 2021 USA	Women 18 years and older Diagnosed with a nonviable intrauterine pregnancy (anembryonic gestation or embryonic/fetal demise) Between 5 and 12 weeks gestation	Diagnosis: incomplete or inevitable miscarriage Women clinically ineligible for EPL medical management
Vejborg, 2007 Denmark	Group A missed miscarriage: a foetus of at least 6 mm (crown rump length; CRL) without cardiac activity (Group A1) or a foetus <6 mm and either no growth or development of cardiac activity over 1 week or declining hCG values (Group A2) or Group B_anembryonic pregnancy: either an empty gestational sac of at least 18 mm (Group B1) or an empty gestational sac of <18 mm with no growth over at least 7 days or a declining hCG value (Group B2)	Twin pregnancies
Wada, 2021 Japan	Patients who had spontaneously or artificially aborted before 22 weeks of gestation RPOC diagnosis was made by ultrasound as follows: (1) an intrauterine high‐echoic or heterogenetic lesion adjacent to the myometrium, (2) a hypervascular lesion in the uterine cavity	

Abbreviations: CRL, crown rump length; ED, emergency department; EFD, early fetal demise; EMV, enhanced myometrial vascularity; EPL, early pregnancy loss; Hb, hemoglobin; LMP, last menstrual period; MSGD, mean gestational sac diameter; NSAID, non‐steroidal anti inflammatory drug; NT, nuchal translucency; PSV, peak systolic velocity; RPOC, retained products of conception; TVS, transvaginal scan.

**TABLE 4:** QUIPS tool quality assessment table: rating of risk of bias (high/moderate/low).

Author, year	Study participation	Study attrition	Prognostic factor measurement	Outcome measurement	Study confounding	Statistical analysis and reporting
Country						
Acharya, 2002 UK	Low	Low	Low	Low	Low	Moderate
Banerjee, 2013 UK	Low	Low	Low	Low	Low	Moderate
Casikar, 2013 Australia	Low	Low	Low	Low	Low	Low
Casikar, 2013b Australia	Low	Low	Low	Low	Low	Low
Casikar, 2010 Australia	Low	Moderate	Moderate	Low	Low	Low
Casikar, 2012 Australia	Low	Moderate	Low	Low	Moderate	Low
Creinin, 2006 USA	Low	Low	Low	Low	Low	Low
Elson, 2005 UK	Low	Low	Low	Low	Low	Low
Fernlund, 2020 Sweden	Low	Moderate	Low	Low	Moderate	Low
Grewal, 2019 UK	Moderate	Low	Moderate	Moderate	Moderate	Low
Guedes‐Martins, 2015 Portugal	Moderate	Low	Moderate	Moderate	Moderate	Low
Hamel, 2022 Netherlands	Low	Low	Low	Low	Low	Low
Lavecchia, 2015 Canada	Moderate	Moderate	Moderate	Low	Low	Moderate
Luise, 2002 UK (BMJ)	Low	Moderate	Low	Low	Low	Low
Luise, 2002 UK (UOG)	Low	Moderate	Low	Low	Low	Low
Lusink, 2018 Australia	Moderate	Low	Low	Low	Moderate	Low
Sairam, 2001 UK	Moderate	Moderate	Low	Low	Moderate	Low
Sajan, 2020 India	Moderate	Low	Moderate	Low	Low	Low
Schreiber, 2015 USA	Moderate	Low	Moderate	Moderate	Low	Moderate
Schwarzler, 1999 UK	Moderate	Low	Moderate	Moderate	Moderate	Moderate
Sonalkar, 2021 USA	Low	Moderate	Low	Low	Moderate	Low
Trinder, 2006 UK	Low	Low	Low	Low	Low	Moderate
Vejborg, 2007 Denmark	Low	Moderate	Low	Low	Moderate	Low
Wada, 2021 Japan	Moderate	Moderate	Moderate	Low	Low	Moderate

### 3.1 Search results

After the initial search through PubMed, Medline, and Google Scholar, a total of 2311 records were screened. Following the initial screen, 2124 studies were excluded due to the title alone, and 187 abstracts were retained and examined. Of those, 124 were excluded because they were not relevant to the research questions, were not available in English, or were duplicate studies. By duplicate studies, we refer to the retrieval of the same study through different databases. Of the remaining 63 full‐text publications that were examined, 39 were review articles or did not evaluate specific predictors of miscarriage management with a comparative cohort. They were therefore excluded, leaving 24 studies for inclusion in the review.^3^, ^6^, ^15^, ^16^, ^17^, ^18^, ^19^, ^20^, ^21^, ^22^, ^23^, ^24^, ^25^, ^26^, ^27^, ^28^, ^29^, ^30^, ^31^, ^32^, ^33^, ^34^, ^35^, ^36^ These studies ranged in publication date from 1999 to 2022. An overview of the search results and screening process is summarized in the study flow diagram (Figure 1).

### 3.2 Study characteristics

Data from the 24 studies included in this systematic review demonstrated considerable variation concerning the research question with regards to the predictive parameters chosen to evaluate, the method of miscarriage management, and the outcomes defined as successful management. There was one randomized controlled trial identified by the search.^34^ All other relevant studies were either retrospective or prospective cohort studies.

14 of the identified studies evaluated expectant management, and 11 studies evaluated medical management (with one study looking at both). The follow‐up cutoff point varied from 3 days to 8 weeks, and the regime of medical management varied across the studies. The number of patients included in analysis varied between studies, ranging from 31 to 451 (Table 1).

### 3.3 Demographic and patient history variables

#### 3.3.1 Age

Four studies evaluated maternal age as a predictor of miscarriage management outcome.^18^, ^22^, ^31^, ^33^ Of these studies only Casikar et al.^18^ found lower maternal age to be statistically significant in predicting expectant management success (*p* = 0.0250) in the training set (*n* = 186) and 0.0110 (*n* = 126) in the test set.

#### 3.3.2 Ethnicity

Schreiber et al.^31^ looked specifically at ethnicity as a predictor and found race was not statistically significant in predicting successful management of miscarriage. However, looking specifically at the Hispanic population compared to non‐Hispanic; the study identified women of Hispanic ethnicity were less likely to succeed with one dose of 800 μg misoprostol (*p* = 0.009). They found that the success and failure groups were otherwise demographically similar. In contrast, Sonalkar et al.^33^ analyzed 297 patients undergoing medical management and found no significant difference in outcomes in the Hispanic population and similarly no difference across the cohort broken down by race. Hamel et al.^6^ included univariate analysis of ethnicity as a predictor of medical miscarriage management, and this was found to be not significant (*n* = 342).

#### 3.3.3 Gestation by dates

Five studies looked at gestational age by patient reported last menstrual period to determine the effect on miscarriage management outcomes.^18^, ^22^, ^23^, ^32^, ^33^ In all of these studies, the chances of miscarriage resolving were independent of gestational age.

#### 3.3.4 Previous delivery type

Casikar et al.,^18^ looked at previous vaginal delivery and previous cesarean section and found both were not predictive of miscarriage management outcomes. Fernlund et al.^23^ analyzed the study group only by previous vaginal delivery and similarly found there was no correlation with expectant management outcomes.

#### 3.3.5 Parity

Five studies analyzed outcomes based on parity.^21^, ^23^, ^28^, ^31^, ^33^ Schreiber et al.^31^ and Creinin et al.^21^ both found a statistically significant impact of parity. Schreiber et al.^31^ found a parity >2 had lower odds of successful pregnancy expulsion with medical management (*p* = 0.010) in a sample size of 95 patients. Creinin et al.^21^ similarly found that patients with nulliparity were significantly more likely to have successful medical management of miscarriage, sample size 485 patients. The other three studies^23^, ^28^, ^33^ found no relationship between parity and miscarriage management outcomes.

#### 3.3.6 Miscarriage history

Three studies analyzed the impact of miscarriage history on miscarriage management outcomes, in all three studies univariate analysis found no significance of miscarriage history as a predictor of miscarriage management outcome.^6^, ^18^, ^31^ Schreiber et al.^31^ looked at number of prior miscarriages with medical management in a study size of 95 patients (*p* = 0.692). Casikar et al.^18^ analyzed the impact of miscarriage history on patients opting for expectant management miscarriage. In the training set (*n* = 186) this feature was significant (*p* = 0.035); however, in the test set (*n* = 126) miscarriage history was not significant on outcomes (*p* = 0.195). Given the test set did not confirm the significance, the study concluded that miscarriage history is not a significant predictor of successful expectant miscarriage management. Hamel et al.^6^ found that for all the following variables, the odds ratio was significant: previous early pregnancy loss, previous medical early pregnancy loss treatment, and previous uterine aspiration.

#### 3.3.7 History of termination of pregnancy

Previous termination of pregnancy was analyzed as a specific risk factor by Casikar et al.^18^ and found not to be significant in the cohort of 312 patients followed for success of expectant management at 2 weeks. Sonalkar et al.^33^ analyzed 297 patients undergoing medical management into those who have previously had an induced abortion or medical abortion or surgical abortion and found none to be significant predictors of medical management of miscarriage success.

#### 3.3.8 Smoking status

Two studies separated analysis of outcomes by maternal smoking status with conflicting results. Casikar et al.^18^ in the analysis of 312 patients did not find smoking status had an impact on expectant management outcome. In contrast, Sonalkar et al.^33^ analyzed 297 patients undergoing medical management and found that non‐smoking status was the only significant predictor of medical management of miscarriage success.

### 3.4 Ultrasound features

#### 3.4.1 Measurements

Seven studies looked at ultrasound measurements as predictors of successfully completing a miscarriage following expectant or medical management.^15^, ^22^, ^23^, ^26^, ^27^, ^28^, ^31^ Three studies analyzed gestational sac measurements and generated conflicting results. Acharya et al.^15^ and Schrieber et al.^31^ found no correlation between gestational sac volume and outcome with expectant and medical management, respectively. In contrast, Lusink et al.^28^ found mean gestation sac diameter to be a significant predictor of successful medical management (*p* = 0.046).

Elson et al.^22^ identified a positive correlation between the diameter of retained pregnancy tissue and expectant management outcome, and Fernlund et al.^23^ found a positive correlation between embryo crown‐rump length and expectant management outcomes. Lavecchia et al.^26^ analyzed medical management outcomes and found that endometrial cavity anterior–posterior diameter measurements were a significant predictor. However, endometrial cavity transverse diameter, longitudinal diameter, and volume were not significant in the prediction of medical management miscarriage outcomes. Luise et al.^27^ analyzed a group of incomplete miscarriages (*n* = 221) and found that there was no statistically significant relationship between the initial presence of a gestational sac or endometrial thickness and the success rate of expectant management.

#### 3.4.2 Type of miscarriage

Ten studies analyzed outcomes of miscarriage management based on type of miscarriage at initial diagnosis.^3^, ^18^, ^19^, ^23^, ^28^, ^29^, ^30^, ^33^, ^34^, ^35^ Amongst these, five studies^18^, ^23^, ^29^, ^30^, ^35^ undertook statistical analysis which demonstrated that the type of miscarriage had a significant correlation with the likelihood of success of expectant/medical management. Casikar et al.^19^ found differing rates of success based on miscarriage type with expectant management after 2 weeks: Incomplete miscarriage 71% (*n* = 130), empty sac 53% (*n* = 36), and missed miscarriage 35% (*n* = 37). Luise et al.^3^ similarly found differing rates of success: Incomplete miscarriage 84% (*n* = 221), empty sac 52% (*n* = 92), and missed miscarriage 59% (*n* = 138). Trinder et al.^34^ is the only randomized controlled trial identified and included in this systematic review; the patients were randomized to different management types. The cohorts were then analyzed separately in two groups based on type of miscarriage: early fetal demise and incomplete miscarriage. In the expectant group 50% women with early fetal demise required surgery later (*n* = 306) and 25% with incomplete miscarriage required surgery later (*n* = 92). In the medical management group 38% women with early fetal demise required surgery later (*n* = 308) and 29% with incomplete miscarriage required surgery later (*n* = 26). Two studies^28^, ^33^ determined that there was no significant relationship between the type of miscarriage and miscarriage management outcome. There is heterogeneity in how the types of miscarriage are defined, the method of management, and the definition of successful outcome, which likely accounts for the conflicting findings.

#### 3.4.3 Vascularity

Six studies assessed the impact of blood flow as a predictor of outcomes.^20^, ^23^, ^24^, ^25^, ^32^, ^36^ Casikar et al.^20^ demonstrated a statistically significant link between qualitative power Doppler scoring and prediction of successful expectant management. Grewal et al.^24^ found enhanced myometrial vascularity was associated with the presence of retained pregnancy tissue, thus an indicator of unsuccessful expectant management. Guedes‐Martins et al.^25^ determined cut‐off points for the uterine artery pulsatility and resistance indices that could discriminate cases more likely to resolve spontaneously. Schwarzler et al.^32^ and Wada et al.^36^ demonstrated that groups with and without vascularity had different outcomes, although analysis found neither to be statistically significant. Schwarzler et al.^32^ is a prospective study assessing color Doppler to determine intervillous flow (*n* = 85). Wada et al.^36^ assessed vascularity in a retrospective cohort of patients with retained pregnancy tissues (*n* = 44). Fernlund et al.^23^ demonstrated no statistically significant correlation between blood flow in presumed intervillous space and expectant management outcome.

### 3.5 Presenting symptoms

#### 3.5.1 Vaginal bleeding

Nine studies examined the relationship between bleeding as a presenting symptom and miscarriage management outcomes.^17^, ^18^, ^21^, ^22^, ^23^, ^28^, ^31^, ^33^, ^36^

Two studies^17^, ^18^ found a statistically significant relationship between vaginal bleeding and successful expectant miscarriage management. In total, these two studies analyzed the outcomes of 522 patients following 2 weeks expectant management and found the outcomes to be significantly different between the symptomatic bleeding cohort and the asymptomatic cohort. One study^21^ found vaginal bleeding within the 24 h prior to medical management was predictive of successful outcome (*n* = 485).

Five studies^22^, ^23^, ^28^, ^31^, ^33^ found no statistically significant correlation between the presence of vaginal bleeding and successful miscarriage management. Two assessed expectant management outcomes^22^, ^23^ and three assessed medical management outcomes.^28^, ^31^, ^33^ In combination these five studies examined the outcomes of 830 cases of miscarriage management. The method of management, definition of vaginal bleeding, and success varied and this subjective presenting complaint did not show any significant correlation with miscarriage management outcomes across the studies.

#### 3.5.2 Lower abdominal pain

Seven studies examined the relationship between abdominal pain as a presenting symptom and miscarriage management outcomes.^17^, ^18^, ^21^, ^23^, ^28^, ^31^, ^33^

Six studies^17^, ^18^, ^23^, ^28^, ^31^, ^33^ did not find a statistically significant relationship between abdominal pain and successful expectant/medical miscarriage management. In combination these six studies examined the outcomes of 1384 cases of miscarriage management. The method of management, definition of abdominal pain, and success varied and this subjective presenting complaint consistently failed to show any significant correlation with miscarriage management outcomes across the studies. Creinin et al.^21^ did report a significant relationship between lower abdominal pain and successful medical management; it is important to note that this study defined significance as a *p*‐value <0.1. Thus, the variable of lower abdominal pain had an association with successful medical management (*n* = 485, *p* = 0.08).

Hamel et al.^6^ grouped vaginal bleeding and abdominal pain together as ‘minor clinical symptoms’. This variable was included in the logistic regression model developed to predict outcome of medical management. The regression co‐efficient value was 0.853 for ‘minor clinical symptoms’ present at the start of treatment as a predictor of medical management success (*n* = 344).

### 3.6 Biochemical markers

#### 3.6.1 Progesterone

Five studies analyzed the differences in serum progesterone between successful and unsuccessful miscarriage management groups.^16^, ^22^, ^23^, ^31^, ^32^

Four studies with a collective total of 276 patients found that measurements of serum progesterone were significantly different between women who experienced successful and unsuccessful outcomes. Banerjee et al.^16^ analyzed 52 patients and found a serum progesterone of <10 nmol/L to be statistically significant in predicting failed medical management of miscarriage. Elson et al.^22^ included 54 patients and identified a statistically significant difference in the value of serum progesterone between the successful and unsuccessful cohorts of expectant management. The successful expectant management cohort had a lower level of serum progesterone. Fernlund et al.^36^ had a total of 85 patients opting for expectant management and similarly found that progesterone levels were statistically significantly lower in the successful expectant management group. Schwarzler et al.^32^ examined the progesterone levels of a total of 85 patients, multivariate analysis alongside human chorionic gonadotrophin (HCG) showed the progesterone coefficient to be 0.159 and statistically significant in predicting successful expectant management (*p* < 0.001).

One study did not find measurements of serum progesterone to be a significant predictor of outcome. Schreiber et al.^31^ undertook a sub‐analysis from a randomized controlled trial including 95 patients and found that a progesterone of <10mmmol/l was not significant in predicting successful medical management of miscarriage.

#### 3.6.2 Human chorionic gonadotrophin

Three studies totaling 224 patients demonstrated that serum beta HCG was significantly different between successful (lower HCG) and unsuccessful (higher HCG) cohorts of miscarriage management studies.^22^, ^23^, ^32^ As with serum progesterone, Schreiber et al.^31^ was the only study that did not find a significant difference in serum beta HCG in the successful medical management group compared to the unsuccessful group.

#### 3.6.3 Other biochemical markers

Elson et al.^22^ further analyzed levels of 17‐hydroxy‐progesterone (17‐OHP), insulin growth factor‐binding protein‐1 (IGFBP‐1), Inhibin A, Inhibn pro aC across 54 patients opting for expectant management. The study demonstrated that 17‐OHP and IGFBP‐1 were not a predictors of miscarriage outcome, whereas Inhibin A and Inhibin pro aC did show statistically significantly differences between successful and unsuccessful outcomes. Low serum inhibin A was associated with successful expectant management, this reflects a low amount of retained functioning trophoblast. Inhibin pro aC is a product of the corpus luteum and has been shown to fall after mifepristone for medical termination of pregnancy.^37^

Schreiber et al.^31^ analyzed biomarkers in 95 women opting for medical management, they found no difference in Activin A, glycodelin, human placental lactogen, or estradiol level. The only statistically significant biomarker was ADAM‐12 in this study. ADAM‐12 is expressed in placental tissue and provokes myogenesis, thus is proposed to have a role in placenta development.

## 4 DISCUSSION

This is the first systematic review to address predictors of the success of expectant and medical management of miscarriage. The search identified a number of variables that have been investigated as predictors of miscarriage management outcome, which can be broadly divided into: (1) Demographics and patient history variables. (2) Ultrasound features of the pregnancy or retained pregnancy tissue. (3) Presenting symptoms. (4) Biochemical markers.

The main strength of this study is the scope of variables included to determine all previously investigated predictive factors that correlate to expectant or medical miscarriage management outcomes. Other strengths include the robust bias ascertainment methodology and the large number of participants included.

The analysis demonstrated a high level of heterogeneity, as the studies included varied in definition of miscarriage management success, parameters of the management undertaken, and how predictors were described. For this reason, meta‐analysis across these studies was not possible. However, this systematic review does demonstrate that in specific circumstances there is evidence that particular variables predict miscarriage management outcomes. The heterogeneity of definitions across the studies outlines the need for an international collaboration to form a consensus on definitions for future miscarriage management studies.

Analysis of the individual predictors in this systematic review showed that the highest number of studies analyzed ultrasound features as a predictor of outcomes. Nine studies analyzed type of miscarriage as a predictor. Incomplete miscarriage indicates that the process of spontaneous resolution has begun or, for some reason has been halted midway. Therefore, the conflicting outcomes may be a result of the time point at which the scan was undertaken. Similarly, missed miscarriage diagnosis does not take into account at what point the pregnancy stopped developing, a pregnancy that has had no growth over 2 weeks compared to a pregnancy that has just stopped developing may have different outcomes. The size of pregnancy relative to the expected gestation by dates is examined by Casikar et al.,^19^ as they excluded women who were diagnosed with a missed miscarriage when the crown‐rump length was ≥3 weeks smaller than the gestational age based on last menstrual period.

The complex invasive interaction between pregnancy tissue and maternal tissue is likely to have a role to play in defining which cases have successful expectant and medical management.^38^ Thus, those with missed miscarriage smaller than the gestation age by last menstrual period, prolonged incomplete miscarriage, and retained pregnancy tissue; physiologically have different processes at play that will impact management outcomes.

The demographic variables explored include age, ethnicity, and smoking status, all of which have been linked to risk of early pregnancy loss.^1^, ^39^, ^40^, ^41^, ^42^ Therefore, it is reasonable to consider that these variables may in turn influence the success for expectant and medical management. The history of termination of pregnancy and parity were also considered as predictors.^43^ A correlation between previous uterine aspirations as a predictor for unsuccessful treatment may reflect that these individuals have already had previous failed medical/expectant management attempts, further to this it is known that cervical dilatation can lead to damage and scarring of the cervical tissue, which may prevent cervical weakening in future medical/expectant management of miscarriage, leading to lower chance of success in early pregnancy losses.

The presenting symptoms explored as predictors were vaginal bleeding and pain at presentation. Similar to the concept of ultrasound features as a predictive indicator of success, these symptoms may indicate the start of the spontaneous resolution, which therefore would make success by expectant or medical more likely; or these symptoms may have been ongoing for a while indicating that the pregnancy has been disrupted but is unlikely to resolve without surgical intervention. The nuances of how these symptoms are recorded may be influential in determining whether they predict the likely or unlikely resolution of early pregnancy loss by expectant or surgical management.

Progesterone and HCG are well established biomarkers in the history of early pregnancy.^43^, ^44^ There is evidence to support the use of progesterone supplements in IVF^45^, ^46^ and more recently in miscarriage risk reduction.^47^ Falling HCG levels have been established as predicting miscarriage^43^; therefore, these levels may correlate with the timeline of when miscarriage has occurred and thus indicate potential success of management options. Other biomarkers evaluated included: Inhibin A, Inhibin pro aC, and ADAM 12. The pathophysiology of miscarriage is complex and multifactorial, and thus it stands to reason that a number of biomarkers may predict miscarriage management outcomes to varying degrees in specific circumstances. A multifactorial analysis of predictors is needed to determine individualized outcomes and guide patient choice.

Having a prediction tool that would enable clinicians to guide patients away from expectant or medical management if it is unlikely to be successful would offer significant benefits. The short‐term national economic cost of miscarriage has been estimated to be approximately £471 million (equivalent to 556 million euros) per year in the UK.^1^ While more research is needed to fully understand the economic implications, addressing the psychological and emotional well‐being of those affected is crucial. Miscarriage can trigger a mourning process similar to that experienced after death of a loved one.^12^ For some individuals, the grief may persist for months or even years, affecting their emotional well‐being and quality of life.^48^ The process of additionally dealing with the decision surrounding management choices is likely an additional stressor,^49^, ^50^ which may have a longer‐term impact on the psychological sequalae of miscarriage.^51^ Optimizing the counseling of women through personalized guidance will have a positive impact.

## 5 CONCLUSION

We have demonstrated that a range of predictors are associated with the outcome of expectant and medical miscarriage management. Relevant predictors include demographics, ultrasound features, presenting symptoms, and biochemical markers. Across the 24 studies there is marked heterogeneity in the definition of miscarriage, predictors, and management outcomes. Although associations with certain variables and miscarriage management outcomes are described, these are not consistent across the studies. We conclude that there is evidence supporting the possibility to offer personalized miscarriage management advice with case specific predictors. Further larger studies with consistent definitions of predictors, management, and outcomes are needed in order to better support women through the decision‐making of miscarriage management.

## AUTHOR CONTRIBUTIONS

All authors developed the strategy for the literature search, reviewed the outputs of the searches, and reviewed and approved the manuscript. Sughashini Murugesu and Tom Bourne wrote the drafts and guided the development of the article.

## FUNDING INFORMATION

Imperial Health Charity/NIHR BRC Grant RFPR2324_16.

## CONFLICT OF INTEREST STATEMENT

There are no conflicts of interest to declare.
